# Ferredoxin Containing Bacteriocins Suggest a Novel Mechanism of Iron Uptake in *Pectobacterium* spp

**DOI:** 10.1371/journal.pone.0033033

**Published:** 2012-03-09

**Authors:** Rhys Grinter, Joel Milner, Daniel Walker

**Affiliations:** 1 Institute of Infection, Immunity and Inflammation, College of Medical, Veterinary and Life Sciences, University of Glasgow, Glasgow, United Kingdom; 2 Institute of Molecular Cell and Systems Biology, College of Medical, Veterinary and Life Sciences, University of Glasgow, Glasgow, United Kingdom; Vrije Universiteit Brussel, Belgium

## Abstract

In order to kill competing strains of the same or closely related bacterial species, many bacteria produce potent narrow-spectrum protein antibiotics known as bacteriocins. Two sequenced strains of the phytopathogenic bacterium *Pectobacterium carotovorum* carry genes encoding putative bacteriocins which have seemingly evolved through a recombination event to encode proteins containing an N-terminal domain with extensive similarity to a [2Fe-2S] plant ferredoxin and a C-terminal colicin M-like catalytic domain. In this work, we show that these genes encode active bacteriocins, pectocin M1 and M2, which target strains of *Pectobacterium carotovorum* and *Pectobacterium atrosepticum* with increased potency under iron limiting conditions. The activity of pectocin M1 and M2 can be inhibited by the addition of spinach ferredoxin, indicating that the ferredoxin domain of these proteins acts as a receptor binding domain. This effect is not observed with the mammalian ferredoxin protein adrenodoxin, indicating that *Pectobacterium spp.* carries a specific receptor for plant ferredoxins and that these plant pathogens may acquire iron from the host through the uptake of ferredoxin. In further support of this hypothesis we show that the growth of strains of *Pectobacterium carotovorum* and *atrosepticum* that are not sensitive to the cytotoxic effects of pectocin M1 is enhanced in the presence of pectocin M1 and M2 under iron limiting conditions. A similar growth enhancement under iron limiting conditions is observed with spinach ferrodoxin, but not with adrenodoxin. Our data indicate that pectocin M1 and M2 have evolved to parasitise an existing iron uptake pathway by using a ferredoxin-containing receptor binding domain as a Trojan horse to gain entry into susceptible cells.

## Introduction

Bacteriocins are highly potent narrow-spectrum antibacterial protein toxins produced by a variety of Gram-negative bacteria that are active against bacteria closely related to the producing strain [1]. The best characterised of the bacteriocins are the colicins from *E. coli* and genes encoding putative bacteriocins with cytotoxic domains highly homologous to cytotoxic domains of the colicins can be identified in the genomes of a wide variety of Gram-negative bacteria. The cytotoxic activity of colicin-like bacteriocins is housed in a C-terminal domain, with central and N-terminal domains encoding receptor-binding and translocation functions [2]. Colicin cytotoxic domains take the form of a specific nuclease domain that hydrolyses DNA, tRNA or 16S rRNA, a pore-forming domain that depolarises the cytoplasmic membrane, or as in the case of colicin M, inhibits cell wall production through degradation of undecaprenyl-phosphate-linked peptidoglycan precursors [3], [4]. The C-terminal domain is also the site of binding for a specific immunity protein that protects the producing cell from the lethal effects of the toxin [5], [6]. To gain entry into target cells, colicins initially bind to a specific outer membrane receptor and cross this membrane through recruitment of host proteins of the TolABQR-Pal or TonB-ExbBD complexes in the periplasmic space [7], [8]. A number of the receptors for colicins and for the closely related pyocins are TonB dependent with a normal physiological role in iron siderophore uptake [9], [10]. Unlike the colicins, little is known about the receptors and mechanisms of entry used by bacteriocins from plant pathogenic bacteria.

The genus *Pectobacterium* contains necrotrophic plant pathogens, characterised by their ability to secrete cell wall degrading enzymes including pectinases, cellulases, proteases and xylanases. These enzymes are produced in greater abundance in *Pectobacterium* spp. than in other phytopathogenic bacteria and give the genus its distinctive soft rot phenotype. The genus *Pectobacterium* is divided into four species *atrosepticum*, *betavasculorum*, *carotovorum and wasabiae* [11], [12]. Strains of *carotovorum* and *wasabiae* have a broad host range, while *atrosepticum* and *betavasculorum* are restricted to potato and sugar beet respectively [13]. *P. atrosepticum* is the causative agent of black leg in potato, one of the most economically important diseases of any temperate crop [14].

Iron acquisition mechanisms of *Pectobacterium* have not yet been studied extensively, however the genus has been shown to acquire iron by diverse mechanisms including siderophore, haem iron and ferric-citrate production/absorption [15], [16]. The closely related soft rot pathogen *Dickea dadantii* (formerly *Erwinia chrysanthemi*) has been shown to possess two high affinity iron siderophores (achromobactin and chrysobactin) and mutants impaired in the production of these siderophores are less virulent, suggesting iron acquisition is an important virulence determinant in infection [17]. Additionally, transcription of pectolysin genes responsible for degradation of the host cell wall during infection, has been shown to be triggered by iron limitation, suggesting a link between iron limitation and pathogenesis [18].

Plant-type ferredoxins are a super family of proteins containing a single [2Fe-2S] cluster. They are predominantly present in the chloroplasts, where they primarily function as electron carriers from photosystem one to enzymes responsible for carbon, nitrogen and sulphur assimilation [19]. Plant-type ferredoxins are also present in the non-photosynthetic tissues of plants suggesting a wider physiological relevance. Genes very closely related to those encoding plant-type ferredoxins are also present, seemingly uniquely among heterotrophic bacteria, in the genomes of a number of *Pectobacterium* species. These genes were likely acquired by horizontal gene transfer [20]. [2Fe-2S] ferredoxins more distantly related to plant-type ferredoxins are widely distributed through prokaryotic and eukaryotic kingdoms [21].

In this work we have purified and characterised two novel bacteriocins from *Pectobacterium* species. These bacteriocins, named pectocin M1 and M2, contain a predicted N-terminal domain with high levels of sequence similarity to plant ferredoxins and a C-terminal domain highly homologous to the catalytic domain of the *E. coli* bacteriocin, colicin M. The cytotoxic activity of these bacteriocins is limited to *Pectobacterium* spp. and is dependent on iron availability. Our data indicate that the ferredoxin domain acts as a receptor binding domain and that these bacteriocins have evolved to gain entry into susceptible cells through parasitisation of a previously unreported iron uptake system in *Pectobacterium* spp.

## Results

### Identification of pectocin M genes in the genomes of *Pectobacterium* spp

**Figure 1:**
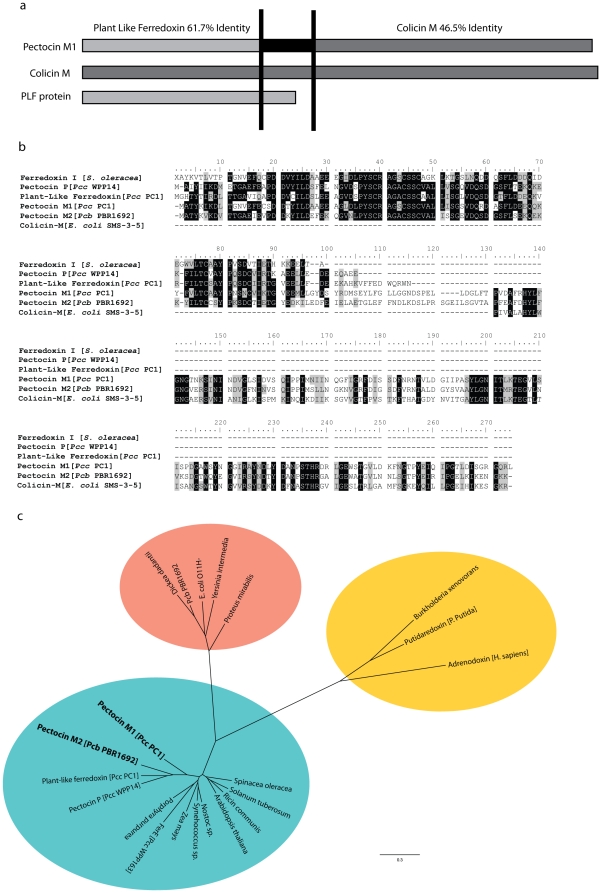
Domain structure, homology and molecular phylogeny of pectocins M1 and M2. a) Domain structure of pectocin M1 and relationship to colicin M and plant ferredoxin. b) Sequence alignment of pectocin M1, M2 and pectocin P (see discussion) with [2Fe-2S] ferredoxin type proteins and colicin M. For clarity of presentation prior to alignment pectocin P was truncated to amino acids 1–101 (N-terminal domain) and colicin M was truncated to amino acids 128–271 (C-terminal domain). Genbank/PDBaccession numbers are as follows: Ferredoxin I [*S. oleracea*] 1704156A, plant-like ferredoxin [*Pcc* PC1] YP_003017870, pectocin M1 [*Pcc* PC1] YP_003017875, pectocin M2 [*Pcb* BPR1692] ZP_03825528, colicin M [*E. coli* SMS-3-5] YP_001739994, pectocin P [*Pcc* WPP14] ZP_03830397. Invariant residues are highlighted in black, residues with similar properties in gray b) Nearest neighbour joining molecular phylogenetic tree of [2Fe-2S] ferredoxins and pectocin ferredoxin domains. Bootstrap values (%) at major nodes are indicated. Species names represent independent ferredoxin proteins from listed species, typifying the class of ferredoxin. Proteins discussed in the study are named with species designation in brackets. Plant ferredoxins and adrenodoxin were aligned with signal peptides removed, pectocin sequences were trimmed to minimum region on homology with plant-like ferredoxin from *Pcc* PC1. Ellipses designate the following: blue = plant-type ferredoxins, red = ferredoxins found predominately in γ-proteobacteria, yellow = ferredoxins involved in electron transport to cytochrome P450. Scale represents substitutions per amino acid site.

As part of a wider study into the identification and potential use of bacteriocins as biocontrol agents we searched the genome sequences of plant pathogenic bacteria for genes encoding putative colicin-like bacteriocins. Two putative colicin M-like bacteriocin genes were identified in the genomes of *P. carotovorum subspp. carotovorum PC1* (*Pcc* PC1) and *P. carotovorum subspp. brasiliensis* BPR1692 (*Pcb* BPR1692). These genes encode proteins that we have named pectocin M1 and pectocin M2, respectively. Pectocin M1 and M2 have an N-terminal domain with approximately 60% identity to spinach ferredoxin I and a C-terminal domain with approximately 46% identity to the catalytic domain of colicin M (Figure 1a and b). The colicin M-like domains of both proteins contain all residues that have previously been shown to be important for the catalytic activity and consequently cytotoxicity of colicin M [22]. A linker region of approximately 20 amino acids, which is not conserved between pectocin M1 and M2 connects the two domains and overall these proteins share 58% sequence identity.

Phylogenetic analysis of the ferredoxin domains of these pectocins show they cluster with a number of other ferredoxin and ferredoxin-domain containing proteins from *Pectobacterium* species (Figure 1c). This cluster of sequences is most similar to [2Fe-2S] ferredoxins from plants and cyanobacteria, with ferredoxins from the genus *Arabidopsis* sharing the highest sequence identity. This cluster is much more distantly related to typical bacterial ferredoxins (Figure 1c). The cysteine residues of plant ferredoxins that coordinate the [2Fe-2S] cluster in the active centre of these proteins are conserved in the pectocins, indicating that these proteins may also contain the [2Fe-2S] cluster (Figure 1b).

### Evolution of the pectocin M1 gene through gene duplication and recombination

**Figure 2:**
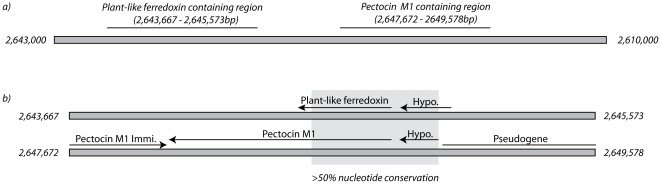
Genomic context of the pectocin M1 gene. a) Position of genomic regions on the chromosome of *Pcc* PC1 containing the pectocin M1 gene and a related plant-like ferredoxin gene. b) Alignment of genomic regions from above, containing the pectocin M1 gene and the related plant-like ferredoxin gene showing annotated open reading frames and nucleotide homology shared between the two regions.

Analysis of the genomic context of pectocin M1 gives clues to its evolutionary origin. As shown in Figure 2a, approximately 3000bp upstream of the gene coding for pectocin M1, is a predicted open reading frame encoding a plant-like ferredoxin with 52% amino acid identity to the ferredoxin domain of pectocin M1. Alignment of the nucleotide sequences of these regions (Figure 2b) shows high nucleotide conservation (>50%) that encompasses the area of sequence similarity between pectocin M1 and the plant-like ferredoxin, as well as part of a hypothetical open reading frame present in both regions, but truncated in the pectocin M1 region. High levels of similarity and truncation of the adjacent open reading in the pectocin M1 region strongly suggests a gene duplication event. Evolution of the gene encoding the active bacteriocin is therefore likely to have occurred after this gene duplication event, through recombination between the duplicated ferredoxin gene and an ancestral bacteriocin carrying a colicin M-like cytotoxic domain. An open reading frame directly upstream of the pectocin M1 gene encodes a likely pectocin M1 immunity protein, which shares 24% amino acid identity with the immunity protein of colicin M. Pectocin M2 does not share this genomic context, however the open reading frame directly upstream codes for a bacteriocin closely related to carocin S2 [23], suggesting it may have been recruited to a genomic island.

### Purification and characterisation of pectocin M1 and M2

**Figure 3:**
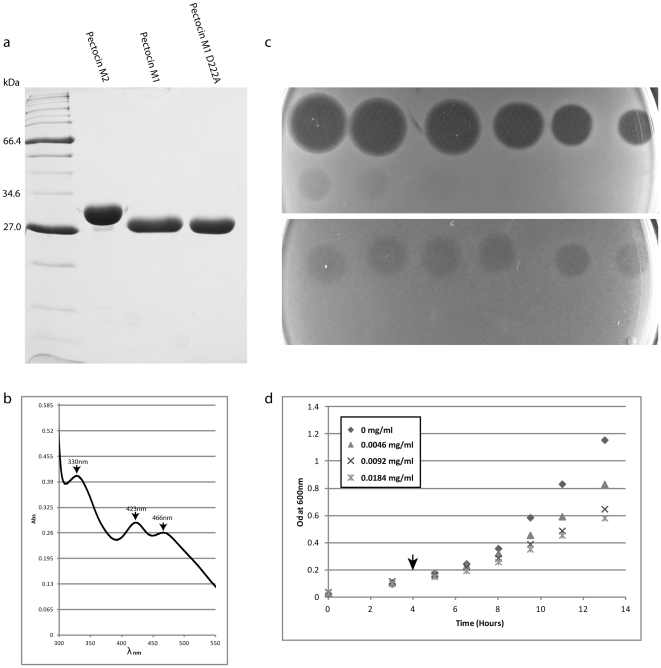
Purification and characterisation of pectocin M proteins. a) SDS PAGE of purified pectocin M2, M1 and M1 D222A b) Absorbance spectrum of pectocin M1 at a concentration 1.2 mg ml^−1^. Maxima at 330, 423 and 466 nm, identical to those observed in plant ferredoxins, indicate the presence of a [2Fe-2S] cluster in pectocin M1. Spectra with identical absorbance peaks were obtained for the pectocin D222A mutant and pectocin M2. c) Agar overlay spot tests of a 3-fold serial dilution (68 µM-0.385 nM) of pectocin M1 spotted onto overlay of *P. atrosepticum* LMG 2386 cells grown in the presence (top) and absence (bottom) of the iron chelator 2,2′-bipryidine (200 µM). d) Liquid growth inhibition assay, test strain LMG 2386, grown in LB broth with 200 µM 2,2′-bipyridine. Purified PM1 was added when indicated.

Pectocin M1 and M2 were expressed in *E. coli* BL21 (DE3) and purified by anion exchange chromatography and gel filtration to >90% homogeneity based on analysis by SDS PAGE (Figure 3a). The purified recombinant proteins were red-brown in colour and the absorption spectra of both proteins displayed maxima at 330 nm, 423 nm, and 466 nm (Figure 3b), which are characteristic of plant ferredoxins [21]. These data show both pectocins M1 and M2 contain a [2Fe-2S] cluster.

Initially the killing spectrum of pectocin M1 and pectocin M2 was tested against five *P. atrosepticum* and five *P. carotovorum* isolates using the agar overlay spot test method on LB agar. Under these experimental conditions pectocin M1 was found to be active against three *atrosepticum* strains and one *carotovorum* strain. The zones of inhibition in this experiment while distinct, were hazy (Figure 3c). Pectocin M2 did not show activity against any of the strains tested under these conditions. Since a number of bacteriocins utilise outer membrane receptors involved in iron uptake [2] we tested the activity of the pectocins under iron limiting conditions induced by addition of the iron chelator 2,2′-bipryidine to the LB agar. Under these conditions, the activity of pectocin M1 was greatly enhanced (Figure 3c), with seven of ten *Pectobacterium spp.* being inhibited. For pectocin M2, three of the ten strains were weakly inhibited (Table 1, Table S1). The minimum inhibitory concentration of pectocin M1 under iron-limiting conditions was calculated using the above method with serial dilutions of pectocin M1 and varied from 14.5–145 nM among susceptible strains. The cytotoxic effect of pectocin M1 in liquid culture was tested by adding varying concentrations of pectocin M1 to an iron limited log-phase culture of the susceptible strain *P. atrosepticum* LMG 2386. A concentration-dependent reduction in growth was observed upon the addition of pectocin M1 (Figure 3d). To determine if pectocin M1 and M2 might be active against more distantly related bacterial species, pectocin M1 and pectocin M2 were tested for inhibitory activity against strains of *E. coli*, *Pseudomonas syringae*, *Pseudomonas aeruginosa* and *Erwinea rhapontici* (see Table S2). None of these more distantly related bacteria showed any susceptibility to pectocin M1 or M2 (at 1.2 and 10 mg/ml respectively) suggesting that the activity of these pectocins is limited species of *Pectobacterium* closely related to the producing strain.

**Table 1:** Susceptibility of *Pectobacterium* strains to pectocin M1 and M2.

Species		No. Strains susceptible to indicated Pectocin	
	*No. Strains tested*	*M1*	*M2*
*Pb. atrosepticum*	5	5	1
*Pb. cartovorum*	5	2	1

### The ferredoxin domain of pectocins M1 and M2 mediate receptor binding

**Figure 4:**
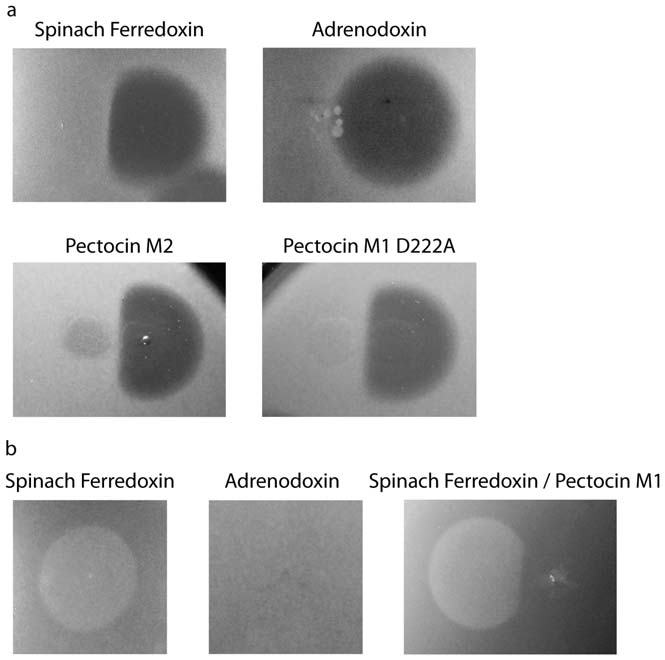
Asymmetric pectocin M1 spot test inhibition zones and growth enhancement due to plant-like ferredoxin, but not adrenodoxin a) Asymmetric pectocin M1 inhibition zones against *P. atrosepticum* LMG2386 due to plant-like ferredoxin containing proteins. Protein concentration for spots were as follows: pectocin M1 1.2 mg ml^−1^, spinach ferredoxin 20 mg ml^−1^, adrenodoxin 30 mg ml^−1^, pectocin M2 10 mg ml^−1^, pectocin M1 D222A 5.6 mg ml^−1^ b) Growth enhancement of *P. carotovorum subspp. carotovorum* LMG 2410 by plant-like ferredoxin containing proteins. Protein concentrations for spots were as follows: spinach ferredoxin 20 mg ml^−1^, adrenodoxin 30 mg ml^−1^, Spinach ferredoxin 20 mg ml^−1^ :pectocin M1 1.2 mg ml^−1^. Pectocin M1 causes asymmetry in zone of enhancement due to spinach ferredoxin in LMG 2410 (400 µM 2,2′-bipyridine) presumably through competition for a common receptor. Pectocin M1 does not enhance growth of this strain under these conditions.

As ferredoxin is a potential iron source for phytopathogenic *Pectobacterium* species we hypothesised that pectocins M1 and M2 were parasitising an existing iron uptake system by using their ferredoxin domain to bind to a cell surface receptor, which has a normal physiological role in iron acquisition from ferredoxin. If this is the case, the addition of a plant ferredoxin at sufficient concentration should abolish pectocin M1 binding to its receptor and therefore abolish its cytotoxic activity. To test this hypothesis spinach ferredoxin was spotted adjacent to pectocin M1 in an agar overlay spot test. Clear inhibition of cell killing was observed in the region where the diffusion zone of spinach ferredoxin overlaps with the pectocin M1 diffusion zone (Figure 4a). Indeed, for all 7 susceptible strains cytotoxicity of pectocin M1 could be abolished to a similar extent by the addition of spinach ferredoxin. This effect was not observed with adrenodoxin, a more distantly related mammalian [2Fe-2S] cluster containing ferredoxin, indicating a level of specificity for plant ferredoxins (Figure 4a). Additionally, in five pectocin M1 susceptible strains that are not sensitive to pectocin M2 the cytotoxicity of pectocin M1 could be abolished by the addition of pectocin M2, indicating these two bacteriocins utilise the same receptor.

To determine if the activity of the colicin M-like domain of pectocin M1 is essential for its cytotoxicity, we made a mutant protein in which Asp222 is replaced by Ala. The equivalent Asp of colicin M has been shown to be essential for the catalytic activity and consequent cytotoxicity of colicin M [22]. Purified pectocin M1 Asp222Ala had no detectable cytotoxicity, but was able to abolish cytotoxicity of wild-type pectocin M1 indicating that it is fully functional in binding to its receptor (Figure 4a). Taken together, these data indicate that receptor recognition occurs through the ferredoxin domain of pectocin M1 and M2 and that the normal physiological role of pectocin M1/M2 receptor is to bind plant ferredoxins.

### Growth enhancement under iron limiting conditions by pectocins and spinach ferredoxin

During spot tests on iron limiting media to determine the killing spectrum of pectocin M1 and M2 we observed that the growth of a number of insensitive *Pectobacterium* strains was observably enhanced where the pectocins were spotted onto the plate. Additionally, some strains that were inhibited by pectocin M1 displayed a zone of enhanced growth peripheral to the zone of inhibition. Four and five of the ten *Pectobacterium* isolates exhibited enhanced growth in the presence of pectocin M1 and M2 respectively. The enhancement of growth due to pectocin M1 was significantly more pronounced than that due to pectocin M2, with strongest enhancement present at 200 µM 2,2′-bipyridine for both pectocin M1 and pectocin M2 (Table S1). This observation led us to hypothesise that *Pectobacterium* strains are able to utilise these ferredoxin domains as an iron source under iron limiting conditions.

To test this idea further, and to test the specificity of this growth enhancement, related plant and mammalian ferredoxin-type proteins (spinach ferredoxin I and human adrenodoxin) as well as syringacin M, a colicin M homologue from *Pseudomonas syringae* which contains an active colicin M catalytic domain but an unrelated N-terminal region [24] (unpublished data) were tested for their ability to enhance the growth of *Pectobacterium* spp. under iron-limiting conditions. Five strains exhibited strongly enhanced growth due to the spinach ferredoxin at either 20 or 2 mg ml^−1^ (Figure 4b), this enhanced growth was dependent on the concentration of the iron chelator 2,2′-bipyridine, with the strongest enhancement at 400 µM (Table S1). No enhanced growth was observed with the [2Fe-2S] cluster containing protein adrenodoxin at 30 mg ml^−1^ or syringacin M at 5 mg ml^−1^.

## Discussion

In this work we have identified, purified and characterised two novel bacteriocins, pectocin M1 and M2, which are active against selected strains of *Pectobacterium* spp. These bacteriocins share a common domain structure, with an N-terminal ferredoxin domain containing a [2Fe-2S] cluster and a C-terminal cytotoxic domain homologous to colicin M. Activity of pectocin M1 was inhibited by the addition of spinach ferredoxin, which strongly suggests that the ferredoxin domain mediates binding to a receptor on the surface of sensitive *Pectobacterium* strains. The mammalian ferredoxin adrenodoxin was unable to elicit this effect, even at a concentration 15 times higher than the lowest tested spinach ferredoxin concentration. This suggests that the pectocin M receptor is specific for plant-type ferredoxin and is not merely binding due to the presence of an iron sulphur cluster. The inhibition of bacteriocin cytotoxicity as an indicator of competition is well established by previous studies of bacteriocin-receptor interactions in *E. coli*. For example, colicin M, which gains entry to the cell via the ferrichrome receptor FhuA, can be blocked by the addition of ferrichrome, the normal physiological ligand of FhuA. In addition, colicin M is able to block albomycin activity, an antibiotic which also uses FhuA as a receptor [22], [25], [26]. Similarly vitamin B_12_, the substrate of the BtuB receptor, is able to inhibit cytotoxicity of the E group colicins which also bind this receptor [27].

The cytotoxicity and spectrum of killing of pectocin M1 and M2 is greatly enhanced under iron limiting conditions suggesting that production of their receptor is unregulated and that this receptor likely plays a role in iron acquisition. This idea is supported by the observation that some strains of *Pectobacterium* spp. are able to exhibit enhanced growth under iron limiting conditions in the presence of a plant-like ferredoxin, either in the form of spinach ferredoxin or the pectocins themselves. The growth enhancement is specific to the extent that adrenodoxin does not elicit this effect. The enhanced growth observed under iron limiting conditions in the presence of the pectocins and spinach ferredoxin, suggests that the iron contained in plant-like ferredoxins can be specifically utilised by *Pectobacterium* species. Taken together, these data strongly suggest that the pectocin M receptor has a normal physiological role in iron acquisition from plant ferredoxin.

The importance of iron acquisition in bacterial pathogenesis of animals is well established and the ability to acquire sufficient iron often determines the success or failure of an infection [28]. The dynamics of iron availability during bacterial pathogenesis of plants is less well understood, although a number of studies have strongly correlated iron acquisition and virulence *in planta* [17], [18], [29]. During infection, *Pectobacterium* species release large quantities of pectinases and cellulases which macerate host tissues creating the distinctive soft rot associated with the genus. This disease phenotype results in cell lysis and the release of intracellular nutrients for absorption by the bacteria [11]. Since ferredoxin is the major iron containing protein in plants it undoubtedly represents an attractive potential source of iron in an environment of low iron availability [30]. As such, the ability *of Pectobacterium* spp. to acquire iron from ferredoxin may represent an important virulence mechanism, although this remains to be tested.

A number of iron acquisition systems involving absorption from iron containing proteins have been identified in pathogenic bacteria [28]. The best studied system involves the transferrin-binding proteins TbpA and TbpB of *Neisseria spp.* These outer membrane proteins bind transferrin with a preference for the holoprotein, strip the protein of its two iron atoms before releasing the apoprotein into the extracellular environment. These proteins have been shown to be specific for the transferrin of the host and are essential for virulence [31]. Whether the entire ferredoxin protein or simply the iron which it contains is taken into the cell by *Pectobacterium* is currently unknown.

The ferredoxin domain of pectocin M1 is closely related to plant ferredoxins and the pectocin M1 gene has seemingly evolved through duplication of a horizontally acquired plant ferredoxin gene and subsequent recombination with an ancestral colicin M-like bacteriocin. The presence of ferredoxin genes in *Pectobacterium* spp. that appear to have been acquired through horizontal gene transfer from plants has been previously noted [20]. In addition to pectocin M1 and M2, we have identified a further putative bacteriocin that contains an N-terminal ferredoxin domain. Analysis of the genome sequence of *Pectobacterium carotovorum subspp. carotovorum* WPP14 revealed a gene encoding a putative bacteriocin (designated pectocin P, Figure 1b) with an N-terminal ferredoxin domain and a C-terminal domain with 41% identity to the C-terminal 187 amino acids of pesticin, a peptidoglycan degrading bacteriocin with muramidase activity from *Yersinia pestis* [32], [33], [34]. Like colicin M the cellular target of pesticin is located in the periplasm and as such a receptor binding and translocation domain sufficient to deliver a colicin M catalytic domain to its site of action would also be sufficient to deliver the cytotoxic domain of a pesticin-like bacteriocin. Recombination and gene duplication have been well established as evolutionary mechanisms for generating bacteriocins with new specificities [1]. Many of the colicins and related bacteriocins identified to date are mosaics of related receptor binding, translocation and catalytic domains [2], [35]. However, the evolution of ferredoxin containing bacteriocins seems to be unprecedented in that *Pectobacterium* spp. has successfully utilised a horizontally acquired host gene for competition against closely related bacterial strains.

## Methods

### Bacterial strains and media

Strains of *Pectobacterium* spp. used in this study are listed in Table 2. Other bacterial strains are listed in Table S2. All bacterial strains were grown in LB broth with the exception of *Pseudomonas syringae* pathovars which were grown in Kings B Broth. All strains were grown at 28°C, with the exception of *E. coli and P. aeurginosa* which were grown at 37°C. Media was supplemented with ampicillin 100 µg ml^−1^, kanamycin 50 µg ml^−1^, isopropyl-ß-D-thiogalactopyranoside (IPTG) 0.1–1 mM or 2,2′-bipyridine 100–400 µM when required.

**Table 2:** Strains and plasmids used in this study.

Strain or Plasmid	Relevant Characteristic(s)	Source or Reference
***P. carotovorum subspp. carotovorum***		
LMG 2410	Isolated from *Cucumis sativus*	BCCM
LMG 2412	Isolated from *Hyacinthus orientalis*	BCCM
LMG 2442	Isolated from *Brassica oleracea*	BCCM
LMG 2444	Isolated from *Solanum tuberosum* (tuber soft rot)	BCCM
LMG 2913	Isolated from soil	BCCM
***P. atrosepticum***		
LMG 2374	Isolated from Apium graveolens var. dulce	BCCM
LMG 2375	Isolated from *Solanum tuberosum* (tuber soft rot)	BCCM
LMG 2386	Isolated from *Solanum tuberosum* (stem rot)	BCCM
LMG 2391	Isolated from soil	BCCM
SCRI 1043	Isolated from *Solanum tuberosum* (tuber soft rot)	[37]
**Plasmids**		
pJexpress411	Kan^r,^ cloning/expression vector, T7 promoter	this study
pJexpress404	Amp^r,^ cloning/expression vector, T5 promoter	this study
pET21-a(+)	Amp^r,^ cloning/expression vector, T7 promoter	Novagen
pJMPC1	Kan^r,^ pJexpress411 with Pectocin M1 inserted into NdeI/XhoI sites	this study
pJMBPR	Kan^r,^ pJexpress411 with Pectocin M2 inserted into NdeI/XhoI sites	this study
pETMPCI	Amp^r,^ pET21-a(+) with Pectocin M1 inserted into NdeI/XhoI sites	this study
pETMPCID222A	Amp^r,^ pET21-a(+) with Pectocin M1 D222A inserted into NdeI/XhoI sites	this study
pETMBPR	Amp^r,^ pET21-a(+) with Pectocin M2 inserted into NdeI/XhoI sites	this study

BCCM = Belgian Co-ordinated Collections of Micro-organisms, NCPPB = National Collection Plant Pathogenic Bacteria, SCRI = Scottish Crop Research Institute.

### Plasmid construction and DNA manipulation

Plasmids used in this study are outlined in Table 2. pJMPCI and pJMBPR which contain the open reading frames of pectocin M1 and M2 and unique NdeI and XhoI sites at the start codons and directly after the stop codons of the pectocin genes were synthesized by DNA 2.0. Plasmids pETMPCI and pMBPR, used for expression of the pectocin M1 and M2, respectively were created by subcloning the NdeI/XhoI fragments of pJMPCI and pJMBPR into the corresponding sites of pET-21a. The D222A mutant of pectocin M1 was created using the QuikChange Site Directed Mutagenesis Kit (Stratagene) utilising pETMPCI as a template and the following primers, (5′-GCA TCC GCG CAT ATA ACG CTC TGT ATG ATG CTA ATC CG-3′, 5′-CGG ATT AGC ATC ATA CAG AGC GTT ATA TGC GCG GAT GC-3′) to give the D222A pectocin M1 encoding plasmid pETMPCID222A.

### Protein expression and purification

Pectocin M1 and M2 were expressed in *E. coli* BL21 (DE3) carrying the plasmid pETPCI or pETBPR, respectively. Cells were grown at 37°C and protein expression was induced by the addition of IPTG at an OD_600_ of approximately 0.6. Cultures were grown for a further six hours at 28°C. Cells were harvested and resuspended in 50 mM Tris-HCl, pH 7.5, 20 mM NaCl and Complete EDTA free protease inhibitor cocktail tablets (Roche) were added. After disruption by sonication the supernatant was clarified by centrifugation and applied to a DE-52 anion exchanged column (Whatman) equilibrated in 50 mM Tris-HCl, pH 7.5, 20 mM NaCl. Bound protein was eluted with a linear gradient of 20–600 mM NaCl in lysis buffer. Pectocin containing fractions were identified based on colour and analysis by SDS-PAGE. Pectocin containing fractions were pooled and dialysed into 50 mM Tris-HCl, pH 7.5, 20 mM NaCl and loaded onto a Superdex S75 26/60 column, (GE Healthcare). To obtain homogenous protein a final purification step using a Mono Q (GE Healthcare) anion exchange column was employed with bound protein eluted with a linear gradient of 20–700 nM NaCl in 50 mM Tris-HCl, pH 7.5. Fractions were pooled as above and dialysed into 50 mM Tris-HCl, pH 7.5, 100 mM NaCl. Dialysed fractions were aliquoted and frozen at −80°C. The absorption spectrum of purified pectocins was determined using a UV-1700 Pharma Spec, UV-VIS spectrophotometer (Shimadzu). Spinach ferredoxin was obtained from Sigma-Aldrich. Adrenodoxin was provided as a gift from Ms Alette Brinth (University of Glasgow) and was judged as >90% holoprotein, based on the ratio of absorbance at 414 nm and 276 nm.

### Growth inhibition/enhancement assays

Inhibition of growth on solid media was determined using the soft agar overlay method [36]. 100 µl of mid-log phase culture of the test strain was added to 5 ml of 0.6% agar in dH_2_O melted and cooled to 42°C. The molten agar was then overlayed onto LB medium with or without 100–400 µM 2,2′-bipyridine. Purified protein was spotted directly onto the surface of the overlay once solidified. To observe inhibition interference proteins were spotted within the others zone of diffusion on the plate. Plates were incubated for a further 12 hours and zones of inhibition/enhancement observed. For inhibition of growth in liquid culture cells were grown in LB broth with 200 µM 2,2′-bipyridine purified pectocin M1 was added to at mid-log phase and the OD_600_ was monitored throughout the experiment.

### Identification of pectocins and bioinformatic analysis

Putative pectocins were identified by interrogating the National Center for Biotechnology (NCBI) non-redundant protein database with the protein sequence for colicin M from *E. coli*, using the pBlast algorithm. Genomic regions containing proteins of interest were then investigated using the NCBI graphic interface. Sequences were saved in FASTA format and analysed using Bioedit, ClustalW multiple sequence alignment tool and CLC genomics workbench.

## Supporting Information

Table S1 Enhancement/Inhibition of growth of *Pectobacterium spp.* at differing concentrations of 2,2′-bipyridine.
(DOC)

Table S2 Non-*Pectobacterium* species tested for susceptibility to pectocins M1 and M2.
(DOC)
