# Supplementary material for: Ferredoxin Containing Bacteriocins Suggest a Novel Mechanism of Iron Uptake in Pectobacterium spp
Source: PLoS One. 2012 Mar 9;7(3):e33033. doi: 10.1371/journal.pone.0033033 (PMC3302902; doi:10.1371/journal.pone.0033033)
Supplement: Table S1 — Enhancement/Inhibition of growth of Pectobacterium spp. at differing concentrations of 2,2′-bipyridine. (DOC) [file pone.0033033.s001.doc]

**Table S1. Enhancement/Inhibition of growth of *Pectobacterium spp*. at differing concentrations of 2,2’-bipyridine.**

|  | **2,2 Bipryidine concentration** | | | | | | | | | | | | | | | |
| --- | --- | --- | --- | --- | --- | --- | --- | --- | --- | --- | --- | --- | --- | --- | --- | --- |
|  | **0µM** | | **100µM** | | | | **200µM** | | | | **400µM** | | | | | |
|  | **Pectocin M1** | | | | | | | | | | | | | | | |
| LMG2444 | - |  | | E | | ++ | | - | |  | | | - | |  | |
| LMG2374 | I | + | I | | ++ | | I | | +++ | | | I | | ++++ | | |
| LMG2391 |  |  | | I | | + | | I/E | |  | | | I | | ++++ | |
| LMG2442 | - |  | | - | |  | | - | |  | | | - | |  | |
| LMG2410 | - |  | | - | |  | | E | | + | | | - | |  | |
| LMG2412 | I | + | | I/E | |  | | I | | +++ | | | I | | ++++ | |
| SCRI1043 | - |  | | - | |  | | I | | + | | | I | | ++++ | |
| LMG2386 | I | + | | I | | ++ | | I | | +++ | | | I | | ++++ | |
| LMG2913 | - |  | | E | | ++ | | I/E | |  | | | I/E | |  | |
| LMG2375 | I | + | | I | | + | | I | | + | | | I | | ++++ | |
|  |  |  | |  | |  | |  | |  | | |  | |  | |
|  | **Pectocin M2** | | | | | | | | | | | | | | | |
| LMG2444 | - |  | | - | |  | | E | | ++ | | | - | |  | |
| LMG2374 | - |  | | - | |  | | I | | + | | | - | |  | |
| LMG2391 | - |  | | - | |  | | - | |  | | | - | |  | |
| LMG2442 | - |  | | - | |  | | E | | + | | | - | |  | |
| LMG2410 | - |  | | - | |  | | E | | ++ | | | - | |  | |
| LMG2412 | - |  | | E | | ++ | | I/E | |  | | | I | | ++ | |
| SCRI1043 | - |  | | - | |  | | - | |  | | | - | |  | |
| LMG2386 | - |  | | - | |  | | I | | + | | | I | | + | |
| LMG2913 | - |  | | E | | + | | E | | ++ | | | - | |  | |
| LMG2375 | - |  | | - | |  | | - | |  | | | - | |  | |
|  |  |  | | - | |  | | - | |  | | |  | |  | |
|  | **Spinach Ferredoxin** | | | | | | | | | | | | | | | |
| LMG2444 | - |  | | - | |  | | - | |  | | | - | | |  |
| LMG2374 | - |  | | - | |  | | - | |  | | | - | | |  |
| LMG2391 | - |  | | - | |  | | - | |  | | | - | | |  |
| LMG2442 | - |  | | - | |  | | E | | + | | | E | | | ++++ |
| LMG2410 | - |  | | - | |  | | E | | + | | | E | | | ++++ |
| LMG2412 | - |  | | - | |  | | - | |  | | | E | | | ++++ |
| SCRI1043 | - |  | | - | |  | | - | |  | | | - | | |  |
| LMG2386 | - |  | | - | |  | | - | |  | | | - | | |  |
| LMG2913 | - |  | | - | |  | | E | | + | | | E | | | ++++ |
| LMG2375 | - |  | | - | |  | | - | |  | | | - | | |  |

Pectocins or spinach ferredoxin spotted onto soft agar containing designated strain overlayed on LB with varying concentration of 2,2’-bipyridine. I = Inhibition of growth, E = Enhancement of growth, I/E = Inner zone of inhibition surrounded by outer zone of enhancement. + to ++++ represent relative strength of inhibition or enhancement, + = weak enhancement, hazy, small zone of inhibition. ++++ = strong enhancement, clear large zone of inhibition.
