# Supplementary material for: Ferredoxin Containing Bacteriocins Suggest a Novel Mechanism of Iron Uptake in Pectobacterium spp
Source: PLoS One. 2012 Mar 9;7(3):e33033. doi: 10.1371/journal.pone.0033033 (PMC3302902; doi:10.1371/journal.pone.0033033)
Supplement: Table S2 — Non-Pectobacterium species tested for susceptibility to pectocins M1 and M2. (DOC) [file pone.0033033.s002.doc]

**Table S2. Non-*Pectobacterium* species tested for susceptibility to pectocins M1 and M2**

| **Strain** | **Relevant Characterisitc(s)** | **Source or Reference** |
| --- | --- | --- |
| ***E. coli*** |  |  |
| *LF82* | Clinical Isolate | [1] |
| DH5α | *F^-^, φ80dlacZΔM15, Δ(lacZYA-argF)U169, deoR, recA1, endA1, hsdR17(rk^-^, mk^+^), phoA, supE44, λ^-^, thi-1, gyrA96, relA1* | Invitrogen |
| BL21(DE3) | F– *omp*T *hsd*SB(rB–, mB–) *gal dcm*(DE3) | Invitrogen |
| ***Erwinia rhapontici***  LMG 2686 | isolated from *Rheum rhabarbarum* | BCCM |
| ***Pseudomonas aeruginosa*** |  |  |
| *PA01* | Clinical Isolate | [2] |
| *PA14* | Clinical Isolate | [3] |
| *PA62* | Environmental Isolate | [4] |
| *E32* | Environmental Isolate | [4] |
| *MSH3* | Environmental Isolate | [4] |
| ***Pseudomonas fluorescens*** |  |  |
| *PfO-1* | isolated from soil | [5] |
| *Pf-5* | isolated from rhizosphere | [6] |
| ***Pseudomonas putida*** |  | [7] |
| *KT2440* | derived from a toluene-degrading isolate *Pseudomonas putida mt-2* |  |
| ***Pseudomons syringae*** |  |  |
| *pv. tomato DC3000* | isolated from *Solanum lycopersicum* | [8] |
| *pv. tomato NCPPB 1107* | isolated from *Solanum lycopersicum* | NCPPB |
| *pv. tomato NCPPB 2563* | isolated from *Solanum lycopersicum* | NCPPB |
| *pv. tomato NCPPB 3160* | isolated from *Solanum lycopersicum* | NCPPB |
| *pv. coronafaciens LMG 5060* | isolated from *Avena sativa* | BCCM |
| *pv. lachrymans LMG 5456* | isolated from *Cucumis sativus* | BCCM |
| *pv. maculicola LMG 2208* | isolated from *Brassica oleracea* | BCCM |
| *morsprunorum LMG2222* | isolated from *Prunus avium* | BCCM |
| *pv. syringae LMG1247* | isolated from *Syringa vulgaris,* type strain | BCCM |
| *pv. syringae LMG 5082* | isolated from *Zea mays* | BCCM |
| *pv. syringae LMG 5084* | isolated from *Pyrus communis* | BCCM |

BCCM = Belgian Co-ordinated Collections of Micro-organisms, NCPPB =National Collection Plant Pathogenic Bacteria, ATCC = American Type Culture Collection

**References**

1. Boudeau J, Glasser AL, Masseret E, Joly B, Darfeuille-Michaud A (1999) Invasive ability of an Escherichia coli strain isolated from the ileal mucosa of a patient with Crohn's disease. Infect Immun 67 :4499-509
2. Stover CK, Pham XQ, Erwin AL, Mizoguchi SD, Warrener P et al. (2000) Complete genome sequence of *Pseudomonas aeruginosa* PAO1, an opportunistic pathogen. Nature 406: 959-964
3. Lee DG, Urbach JM, Wu G, Liberati NT, Feinbaum RL et al. (2006) Genomic analysis reveals that Pseudomonas aeruginosa virulence is combinatorial. Genome Biol. 7: R90
4. Lee CS, Wetzel K, Buckley T, Wozniak, Lee J (2011) Rapid and sensitive detection of Pseudomonas aeruginosa in chlorinated water and aerosols targeting gyrB gene using real-time PCR. J Appl Microbiol 111: 893-903
5. Silby MW, Cerdeno-Tarraga AM, Vernikos GS, Giddens SR,Jackson RW et al. (2009) Genomic and genetic analyses of diversity and plant interactions of *Pseudomonas fluorescens*. Genome Biol 10: R51
6. Paulsen IT, Press CM, Jacques R, Kobayashi DY, Myers GSA et al. (2005) Complete genome sequence of the plant commensal *Pseudomonas fluorescens* Pf-5. Nat Biotechnol 23: 873-878
7. Nelson KE, Weinel C, Paulsen IT, Dodson RJ, Hilbert et al. (2002) Complete genome sequence and comparative analysis of the metabolically versatile Pseudomonas putida KT2440. Environ Micro 4: 799-808
8. Buell R, Joardar V, Lindeberg M, Selengut J, Paulsen IT et al. (2003) The complete genome sequence of the Arabidopsis and tomato pathogen Pseudomonas syringae  pv. tomato DC3000. Proc Natl Acad Sci U S A 100: 10181-10186
